# Phytochemicals, antioxidant, antinociceptive and anti-inflammatory potential of the aqueous extract of *Teucrium stocksianum* bioss

**DOI:** 10.1186/s12906-015-0872-4

**Published:** 2015-10-07

**Authors:** Syed Muhammad Mukarram Shah, Syed Muhammad Hassan Shah

**Affiliations:** Department of Pharmacy, University of Malakand, Chakdara Dir, KPK Pakistan; Department of Pharmacy, Sarhad University of Science and Information Technology, Peshawar, KPK Pakistan

**Keywords:** *Teucrium stocksianum*, Phytochemicals, Antioxidant, Antinociceptive, Anti-inflammatory

## Abstract

**Background:**

Despite availability of a substantial number of potent synthetic drugs**,** medicinal plants are still playing a key role in the discovery of novel and effective drug molecules. Numerous researchers are focusing on the plant based medicines due to its strong safety profiles. *Teucrium* species exhibit profound antidiabetic, analgesic and spasmolytic activities. The methanolic extract and essential oil of *Teucrium stocksianum* possess strong antinociceptive activity. The aim of the current research study was to determine the phytochemicals, antioxidant, analgesic and anti-inflammatory potential of the aqueous extract of *Teucrium stocksianum* Bioss (AETS).

**Method:**

Phytochemical screening was carried out according to standard procedures. The antioxidant potential of the extract was ascertained with the stable organic free radical (2, 2-diphenyl-1-picryl-hydrazyl). Three different pain models, including acetic acid induced writhing, formalin induced paw licking and tail immersion tests were carried out for the determination of antinociceptive potential, while the anti-inflammatory activity was evaluated through carrageenan induced paw edema test in mice. The antinociceptive and anti-inflammatory potentials of AETS were assessed at 100, 200 and 300 mg/kg body weight, while acute toxicity were observed at 1500 mg/kg body weight in various groups of mice.

**Results:**

Phytochemical screening has shown the occurrence of flavonoids saponins, reducing sugars, terpenoids and tannins. AETS exhibited profound antioxidant activity and has shown maximum activity (60.06 ± 0.846) at 250 μg/ml. In the three pain models AETS displayed marked dose dependent antinociceptive potential. AETS exhibited 63.5, 67.61 and 64 % activity in acetic acid induced, formalin induced paw licking and tail immersion tests respectively. The antinociceptive effect of AETS and reference standard drug Tramadol^R^ was significantly reversed by Naloxone, endorsed the central analgesic potential of AETS. Similarly the extract also reversed the paw edema in dose dependent manner. AETS displayed significant (53.81 %) anti-inflammatory effects at a dose of 300 mg/kg that persisted till 5^th^ h. In acute toxicity test AETS was found safe at 1500 mg/kg body weight.

**Conclusions:**

AETS exhibited profound antioxidant activity. The test sample displayed marked antinociceptive potential in all the test procedures, indicating the peripheral and central analgesic effects of AETS. The plant extract also displayed marked anti-inflammatory activity at all test doses.

## Background

The family Lamiaceae comprises of 220 genera and 4000 species around the world. They are most frequently used in indigenous medicines throughout the globe. Various plants of this genera have been used traditionally for the treatment of various ailments, i.e., *Teucrium polium* Linn is used ethno-medicinally for the treatment of pain associated with pregnancy, flatulence, analgesia, liver disorders, jaundice, coughing and miscarriage [1–4].

A number of compounds have been isolated from different species of *Teucrium.* Montanins A, B, C, D and E were isolated from *Teucrium montanum* [5, 6]. Teucrins A and E have been obtained from *Teucrium chamaedrys* [7]. Compounds isolated from *Teucrium quadrifarium* include, 12-epi-teucvidin, teucvidin, teuflin, teucvidin, 19-acetyl-teuspinin and teuquadrin B were isolated [8]. Coll *et al.* [9], have isolated *neo*-clerodanes, namely 6-acetyl-10-hydroxyteucjaponin B, 11-hydroxyfruticolone, 6-acetylteucjaponin B, 7-β-hydroxyfruticolone and deacetylfruticolone from *Teucrium fruticans*.

There have been reported various biological activities from *Teucrium* species, like antioxidant [10], antiseizure [11], hepatoprotective, antimicrobial, butarylcholine and acetylcholine esterase inhibition activities [12, 13]. *Teucrium stocksianum* is one of the important members of the genus *Teucrium*. The methanolic extract and its subsequent fractions have exhibited strong insecticidal, cytotoxic and phytotoxic potentials [14, 15]. The aqueous extracts are most frequently used in the traditional medicine system for the treatment of various disease conditions. Thus in ordered to evaluate and authenticate the folkloric analgesic effect of the plant we therefore determined the antioxidant, antinociceptive and anti-inflammatory potential of the AETS. Furthermore the current study is the extension of our previous research project in which we have determined the antioxidant and antinociceptive potential of the essential oil and methanolic extracts of the same plant [16, 17].

## Methods

### Plant material

*Teucrium stocksianum* was collected in the month of May 2014 from District Swat (Marghuzar valley) in the province of Khyber Pakhtunkhwa (KPK), Pakistan. The plant was authenticated by Professor Dr. Nisar, Department of Botany University of Malakand, KPK, Pakistan. Plant specimen (voucher No, H.UOM.BG.199) was deposited in University Herbarium for future reference.

### Preparation of plant extract

The whole plant was air dried under shade, cut into small pieces and pulverized to coarse powder. About 100 g of the powdered plant material was stirred with 500 ml distilled water at 95 °C for 25 min. The aqueous extract of *T. stocksianum* (AETS) was filtered through Whatman’s #1 filter paper. The AETS was concentrated on water bath at 45 °C. The concentrated extract was stored in glass container in refrigerator for future use.

### Experimental animal

Male and female, Swiss albino mice of same age having 20–30 g weight, were procured from National Institute of Health, Islamabad Pakistan. All the animals were kept in appropriate cages at standard controlled laboratory condition (23 °C, 12 h light and 12 h dark cycle). Food and water were given to all the experimental animals *ad libitum* during acclimatization period. The food was withheld 18 h prior to experiment from all animals while water was available during this period. The animals were randomly divided into various groups (*n* = 6). The experimental protocol for animal studies was approved by the legal bodies (Ethical committee) of University of Malakand KPK, Pakistan according to the guidelines of Scientific Procedure Issue-1 of Animal bylaws 2008.

### Chemicals and drugs

Tramadol^R^ manufactured by Searle Pakistan Ltd (Trade name Tramal) was procured from local market, Naloxone was purchased from Acent Scientific Company, Diclofenic sodium obtained from Sigma and Aspirin manufactured by Reckitt & Colman was used.

### Statistics and calculations

The data obtained were expressed as mean ± Standard error of mean (SEM) and Standard deviation (S.D) of six animals. One-way analysis of variance (ANOVA) and post hoc Dunnett’s test was applied for the comparison among different groups. Differences with *P ≤* 0.05 and lower between groups were considered significant.

### Acute toxicity

For the determination of possible toxicity of the aqueous extract, acute toxicity test was performed. Animals were divided into four groups of either sex (n = 6) and were treated with 500, 1000 and 1500 mg/kg, i.p. The control group received Normal saline (10 ml/kg). All the animals were continuously observed for any gross effect during first 4 h and then the number of dead animals were counted after 24 h [18].

### Antioxidant potential

#### DPPH (2, 2-diphenyl-1-picryl-hydrazyl) radical scavenging potential

DPPH is a stable organic free radical, mostly used for the determination of radical scavenging potential of extract of phytomedicines. The activity was carried out according to the protocol described by Chou *et al*. [19] with slight modifications. Methanolic solution of DPPH (1 mM) was prepared and 01 ml of this solution was mixed to the calculated volume of the aqueous solution of sample containing 20, 40, 60, 80 and 100 μg of the extract respectively. All the test samples, reference standard and control solutions were incubated for half an hour, at controlled temperature (20–25 °C) in the complete absence of light. Afterwards, samples, control and standard were analyzed at 517 nm and absorbance was recorded. DPPH radical scavenging effect was calculated as the inhibition percentage and was determined by the following equation.

$$ \%\mathrm{R}\mathrm{S}\mathrm{A}=\frac{\mathrm{Control}\ \mathrm{absorbance} - \mathrm{Sample}\ \mathrm{absorbance}\kern0.5em }{\mathrm{Control}\ \mathrm{absorbance}} \times 100 $$

The test for each sample was carried out in triplicate and the results were expressed as mean ± SD. Inhibitory concentration (IC_50_) was determined from the graph of % RSA. Quercetin and Ascorbic acid were used as reference standards.

### Analgesic potential

#### Acetic acid induced abdominal writhing test

Animals were divided into five groups, each containing 06 mice. All the groups received intraperitoneal (i.p) acetic acid solution (10 ml/kg, 0.6 %, i.p) 30 min after dosing with either aqueous extract of *Teucrium stocksianum* (100, 200 and 300 mg/kg) or vehicle (0.9 % saline) or reference standard drug, Aspirin (100 mg/kg). The number of abdominal writhes (constrictions) were recorded after 5 min of acetic acid administration for a period of 20 min. Percent inhibition was determined by comparing the results of aqueous extract with the control group [20].

#### Formalin induced paw licking test

Animals were acclimatized in the observation chamber, 15 min prior to the experiment. Animals were divided into 05 groups (*n* = 6). Test groups were treated with 100, 200 and 300 mg/kg(i.p) aqueous extract of the plant, control group was treated with Normal Saline solution (0.9 %, 10 ml/kg, i.p) while the reference standard group received acetylsalicylic acid (100 mg/kg, s.c). The animals received sub-plantar injection of 20 μl (01 %) formalin solution into the right hind paw, 30 min after treatment. Subsequently, the total number of paw licking were observed during initial 0–5 min (Neurogenic phase) and late 20–30 min (Inflammatory phase) after formalin injection. The experiment was performed under strict condition of no disturbance that may affect the animal’s response.

#### Tail immersion test

In this experiment, Swiss albino mice of either sex (n = 6) weighing 20–30 g, were divided into seven groups. Group A treated with Normal Saline (0.9 %, 10 ml/kg, b.w, i.p), group B, C and D received intraperitoneal injection of 100, 200 and 300 mg/kg, i.p of AETS respectively. Group “E” was treated with Tramadol^R^ (30 mg/kg), served as reference standard drug. Pain was induced in each animal by immersing the tail (2–5 cm) in a pot of water maintained at 54 ± 0.5 °C. The reaction time (in seconds, is the time taken by the animal to withdraw the tail from warm water) was recorded after 30 min of dosing. To avoid the tissue damage, 30 s cut-off time was maintained at 54 ± 0.5 °C. To ascertain the opioids receptors involvement in the mechanism of analgesia of AETS, group “F” received 300 mg/kg AETS, 10 min after subcutaneous injection of naloxone (0.5 mg/kg). Similarly group “G” was treated with Tramadol^R^ (30 mg/kg). The reaction time was observed at 0, 30, 60, 90 and 120 min after drug administration respectively [21].

### Anti-inflammatory potential

#### Carrageenan induced paw edema test

The anti-inflammatory potential of the aqueous extract of *T. stocksianum* was evaluated in mice of either sex (25–30 g). Mice were divided randomly in five groups (n = 06) [22]. Group “I” served as a negative control, treated with 10 ml/kg of 0.9 % Normal Saline solution, group “II” received Aspirin 100 mg/kg (positive control), while group III, IV and V received intraperitoneal injection of aqueous extract of *T. stocksianum* at a dose of 100, 200 and 300 mg/kg, respectively. After 30 min of dosing, each mouse received sub planter injection of freshly prepared carrageenan suspension (0.05 ml of 1 % w/v) in the right hind paw. Subsequently the inflammation induced was measured with plethysmometer (LE 7500 plan lab S.L) after carrageenan injection and at 1 h interval for 5 h. The paw volume of the standard drug and AETS treated animals were compared with the negative control group animals and the percent inhibition of inflammation was calculated at different time intervals, using the following formula;

$$ \mathrm{Percent}\ \mathrm{inhibition} = \mathrm{A}\hbox{-} \mathrm{T}/\ \mathrm{A} \times 100 $$

where A is the average inflammation of control and T is the paw volume of test group [23].

## Results and discussions

Owing to phytochemical constituents synthesized by medicinal plants, the plants are considered a very valuable and rich source to obtain the bioactive molecules. The medicinal value of plants is due to phytochemical constituents, synthesized by medicinal plants. Saponins for instance, exhibit significant antidiabetic, cytotoxic and insecticidal activities [14, 15]. Phytochemical screening of the aqueous extract of *T. stocksianum* showed the presence of flavonoids, saponins, reducing sugars, terpenoids and tannins, while phlobutanins, alkaloids, anthraquinonnes and glycosides were not found (Table 1). In our previous work we determined phytochemicals, total phenolic contents and antinociceptive potential of the methanolic extract of *T. stocksianum* [17]*.* A rich literature study is available showing that decoctions/ aqueous preparations are most commonly used for the treatment of different diseases in the traditional medicinal system. In the current study we therefore evaluated the phytochemical composition, antioxidant, antinociceptive and anti-inflammatory potential of the aqueous extracts of *T. stocksianum.*

**Table 1:** Phytochemical screening of aqueous extracts of *T. stocksianum*

S. No	Phytochemicals	Reagents/chemicals	Observations	Results
1	Flavonoids	NaOH + HCl	Discoloration	**+**
2	Saponins	Distilled water	Frothing	**+**
3	Reducing sugars	Fehling's solution	Orange red precipitation	**+**
4	Phlobutanins	HCL	Red precipitate not found	**−**
5	Terpenoids	CHCl_3_ + H_2_SO_4_	Dark green colouration	**+**
6	Alkaloids	Dragendorff’s	Orange red PPTs was not found	**−**
7	Tannins	Ferric chloride	Dark green colouration	**+**
8	Anthraquinonnes	HCl + CHCl_3_ + NH_3_	Rose pink colour	**−**

Key **+** = Present, **−** = Absent

Antioxidants play a vital role in the control of reactive oxygen species (ROS), produced during cell metabolism. ROS are implicated in the main pathogenesis of diabetes, rheumatic joint pain, atherosclerosis and hypertension [24, 25]. In DPPH radical scavenging assay the AETS displayed excellent activity in a concentration dependent manner. The extract showed maximum potential (60.06 %) at 250 μg/ml. IC_50_ value was 136 μg/ml, as shown in Table 2. The antioxidant potential is mainly due to the presence of phenolic compounds like flavonoids and flavones [26]. The present study confirmed the presence of flavonoids in AETS and it is well documented that flavonoids exhibit significant antioxidant activities (Table 1). Thus it is concluded that the flavonoids might be responsible for antioxidant potential of AETS.

**Table 2:** DPPH radical scavenging potential of aqueous extract *T. stocksianum* (AETS)

Test solutions	Concentration μg/ml			IC_50_ (μg/ml)
Sample/standard	62.5	125	250	
AETS	34.70 ± 1.454	48.66 ± 1.50	60.06 ± 0.846	136
Quercetin	94.86 ± 0.872	95.28 ± 1.24	96.56 ± 1.52	<62.5
Acetic acid	90.62 ± 1.856	92.45 ± 1.07	94.24 ± 1.33	<62.5

Values are expressed as means ± SEM of three replicates

Flavonoids have diversified pharmacological activities. Sannigrahi *et al*; have reported antinociceptive and anti-inflammatory activities of crude flavonoids extracted from *Enhydra fluctuans* using different animal models [27]. Acetic acid induced abdominal writhing test is a non specific procedure for the determination of analgesia [28]. Sannigrahi *et al*. [29], have reported antinociceptive and anti-inflammatory activities of crude flavonoids extracted from Enhydra fluctuans using different animal models. In order to relieve the pain mostly narcotic and nonsteridal anti-inflammatory drugs are used. In acetic acid induced abdominal writhing test the test groups treated with 100, 200 and 300 mg/kg of AETS exhibited profound and dose dependent inhibition of the abdominal writhes. Marked activity (63.5 %) was recorded at 300 mg/kg, as compared to control group. The reference standard drug acetyl salicylic acid produced greater inhibitory effect as compared to the highest dose of AETS, depicted in Table 3. Looking at the results of our previous works it has been found that the AETS possesses less analgesic potential (60.06 % at 300 mg/kg) as compared to the methanolic extract of *T. stocksianum* (83.103 % at 150 mg/kg). This pain paradigm releases arachedonic acid and prostaglandins from tissue phospholipids through cyclooxygenase (COX) pathway and are responsible for inflammation and pain [17, 30]. Those drugs molecules which inhibit the writhes preferably by inhibiting the synthesis of prostaglandins are considered as peripherally acting analgesic drugs [16, 30]. On the basis of our findings it could be recommended that AETS exhibits peripheral analgesic effect that might be mediated by inhibiting, either the response or releases of noxious mediators that leads to sensitization and activation of peripheral nociceptors. Moreover, these findings has been confirmed by our recently published research data, in which the ethyl acetate extract exhibited profound anti-inflammatory potential against prostaglandin E_2_ induced paw edema test model in mice [31].

**Table 3:** Antinociceptive effect of AETS 100, 200 and 300 mg/kg in acetic acid induced abdominal writhing test

Samples	Dose	No of writhes	% inhibition
Normal saline	10 ml/kg	73.16 ± 1.81	–
AETS	100 mg/kg	44.33 ± 1.94	39.40*
	200 mg/kg	36.16 ± 1.57	50.57**
	300 mg/kg	28.5 ± 1.56	61.10**
Acetylsalicylic acid	100 mg/kg	16.0 ± 1.96	78.13**

Percent inhibition was calculated in comparison to control group. The data was analyzed by ANOVA followed by Dunnett’s test. The significant values from control were presented with asterisks i.e., *p ≤ 0.05, **p ≤ 0.01

Formalin induced pain paradigm is a well recommended biphasic procedure for the determination of antinociceptive activity [32]. The first phase is for initial 5 min (neurogenic phase) and the second phase takes 20 to 30 min (inflammatory phase). AETS has significantly decreased the paw licking in both neurogenic and inflammatory phases of formalin induced paw liking test in mice. The extract caused 33.45 and 67.61 % inhibition in phase I and II respectively (Table 4). It is well documented that the centrally acting drugs significantly inhibits both phases while peripherally acting drugs can only inhibits phase II. Thus the antinociceptive potential might be due to involvement of both peripheral and central path ways.

**Table 4:** Effect of AETS 100, 200 and 300 mg/kg in formalin induced paw licking test

Samples	Dose	Phase I		Phase II	
		No of paw licking	% inhibition	No of paw licking	% inhibition
Normal saline	10 ml/kg	49.83 ± 2.05	**–**	41.16 ± 1.95	**–**
AETS	100 mg/kg	42.66 ± 1.87	14.38^n.s^	29.33 ± 2.01	28.74*
	200 mg/kg	36.55 ± 1.94	26.75*	19.33 ± 2.23	53.04**
	300 mg/kg	33.16 ± 2.30	33.45**	13.33 ± 1.99	67.61**
Acetylsalicylic acid	100 mg/kg	43.16 ± 1.66	**–**	5.833 ± 0.94	85.83**

Values are expressed as mean ± SEM. The data was analyzed by ANOVA followed by Dunnett’s test; Percent h Inibition was calculated in comparison to the control group. Asterisks shows the significant values (**p ≤ 0.01 and *p ≤ 0.05) *vs.* control group (n = 6) and n.s shows statistically non significant values

**Fig. 1:**
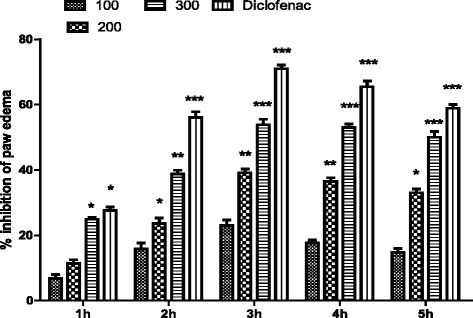
Percent inhibition produced by AETS (100, 200 and 300 mg/kg) of in carrageenan induced paw edema model in mice. Each percent point represents the mean ± SEM for group of 06 mice. Data was analyzed by ANOVA followed by post hoc Dunnett’s test. *Asterisks* show significant values from control. *P <0.05, **P <0.01 and ***P <0.001

To ascertain central analgesic activity we have carried out tail immersion test. Tail immersion test is specific test for the evaluation of central analgesic activity. AETS has significantly (**P <0.01) increased the pain threshold. The extract demonstrated maximum (64 %) antinociceptive activity at 300 mg/kg, indicating the involvement of both spinal and supraspinal path ways of analgesia. The analgesic effect was compared with Tramadol^R^, centrally acting analgesic drug and produce similar analgesic effect like Morphine. Contrary to the achievement of significant threshold (60 min) by AETS, Tramadol^R^ has efficiently and significantly raised the thermal pain threshold within 30 min as shown in Table 5. In order to assess the involvement of opioids receptors in the process, the analgesic effect was antagonized with Naloxone (opioids antagonist). Naloxone significantly reversed the antinociceptive activity of both Tramadol^R^ and AETS, which confirmed the central analgesic activity of AETS (Table 5). In carrageenan induced paw edema test AETS demonstrated a concentration dependent anti-inflammatory activity at all test doses (100, 200 and 300 mg/kg). The anti-inflammatory activity with average volume of paw edema is shown in Table 6 and the percent inhibition of paw edema of aqueous fraction is presented in Fig. 1. The edema created by carrageenan injection was inhibited by the extract in dose dependent manner. A rich literature is available which signify the role of the medicinal plants in the management of inflammation [33]. Numerous researchers have evaluated different classes of phytochemicals for anti-inflammatory activity. Flavonoids mostly exerts anti-inflammatory effect by interfering with arachidonic acid metabolism [34]. Saponins also possess profound anti-inflammatory and cytotoxic activities [35, 36]. The AETS at a doses of 100 and 200 mg/kg displayed 23 and 39 % inhibition of paw edema at 3 h after carrageenan sub planter injection, while 300 mg/kg of AETS displayed marked anti-inflammatory activity that became significant (**P <0.01) at 2 h and remained persistent throughout the procedure, showing maximum effect of 53 % at 3 h after carrageenan sub planter injection. Diclofenac sodium displayed comparatively more significant percent inhibition (71 %, ***P <0.001) of paw inflammation than that of the test doses of AETS of *T. stocksianum* (Fig. 1).

**Table 5:** Effect of AETS 100, 200 and 300 mg/kg in tail immersion test

Samples/standard	Dose mg/kg	Tail withdrawing time in sec				
		0 min	30 min	60 min	90 min	120 min
Normal saline	10 ml/kg	3.35 ± 0.11	3.48 ± 0.16	3.22 ± 0.032	3.39 ± 0.20	3.34 ± 0.19
AETS	100 mg/kg	3.35 ± 0.04^n.s^	3.61 ± 1.03 ^n.s^	4.02 ± 1.03*	3.96 ± 0.4*	3.76 ± 1.04 *
	200 mg/kg	3.36 ± 1.34 ^n.s^	4.19 ± 1.07*	4.55 ± 0.07**	4.48 ± 0.1**	4.20 ± 2.10**
	300 mg/kg	3.37 ± 0.4 ^n.s^	4.80 ± 0.21*	5.36 ± 0.11**	5.30 ± 0.12**	5.03 ± 0.10**
Tramadol^R^	30 mg/kg	3.5 ± 0.20 ^n.s^	4.99 ± 1.33**	5.69 ± 0.02**	5.49 ± 0.06**	5.39 ± 0.05**
Antinociceptive effect of AETS and Tramadol^R^ antagonized by Naloxone						
AETS	300 mg/kg	3.37 ± 1.05 ^n.s^	4.10 ± 2.10 ^n.s^	4.13 ± 1.07 ^n.s^	4.20 ± 1.05 ^n.s^	4.26 ± 1.70 ^n.s^
Tramadol^R^	30 mg/kg	3.38 ± 1.04 ^n.s^	3.75 ± 2.41 ^n.s^	3.72 ± 1.50 ^n.s^	3.75 ± 1.02 ^n.s^	3.70 ± 1.04 ^n.s^

Values expressed as mean ± SEM, The data was analyzed by ANOVA followed by Dunnett’s test, n.s shows statistically non significant values, **p <0.01 and *p <0.05 *vs.* control group (n = 6)

**Table 6:** Concentration dependent anti-inflammatory effect of AETS in carrageenan induced paw edema test

Test sample/drug	Dose mg/kg	1 h	2 h	3 h	4 h	5 h
Normal saline	10 ml	0.220 ± 0.011	0.233 ± 0.84	0.235 ± 1.084	0.236 ± 1.033	0.236 ± 1.02
Diclofenac sodium	10	0.160 ± 0.106	0.101** ± 1.08	0.070** ± 1.028	0.081** ± 1.05	0.096** ± 1.07
AETS	100	0.205 ± 1.084	0.196 ± 0.11	0.181* ± 1.10	0.195* ± 1.30	0.201* ± 1.06
	200	0.195 ± 1.0764	0.178 ± 1.87	0.143** ± 0.98	0.150** ± 0.10	0.158* ± 0.88
	300	0.171* ± 0.79	0.138** ± 0.80	0.106** ± 1.08	0.111** ± 1.10	0.118** ± 0.11

Values are reported as mean ± SEM, n = 06. Data was analyzed by ANOVA followed by post hoc Dunnett’s test. Asterisks show significant values from control. *p <0.01, **p <0.001

## Conclusion

We concluded from the current research work that the aqueous extract of *T. stocksianum* (AETS) possesses marked antioxidant, analgesic and anti-inflammatory potentials. AETS has shown both peripheral and central antinociceptive activity in a dose dependent manner. The extract also significantly inhibited the inflammation induced by carrageenan. All these activities might be attributed to flavonoids and saponins present in AETS. Based on our findings we recommend the plant extract for bioassay guided isolation of the secondary metabolites, to uncover the mechanism of above mentioned activities.
